# Bronchial Epithelial Cells from Cystic Fibrosis Patients Express a Specific Long Non-coding RNA Signature upon *Pseudomonas aeruginosa* Infection

**DOI:** 10.3389/fcimb.2017.00218

**Published:** 2017-05-29

**Authors:** Viviane Balloy, Remya Koshy, Lea Perra, Harriet Corvol, Michel Chignard, Loïc Guillot, Vinod Scaria

**Affiliations:** ^1^Sorbonne Universités, UPMC Univ Paris 06, INSERM, Centre de Recherche Saint-Antoine (CRSA)Paris, France; ^2^GN Ramachandran Knowledge Center for Genome Informatics, CSIR Institute of Genomics and Integrative BiologyDelhi, India; ^3^Pneumologie Pédiatrique, AP-HP, Hôpital TrousseauParis, France; ^4^CSIR Institute of Genomics and Integrative Biology, Academy of Scientific and Innovative ResearchDelhi, India

**Keywords:** cystic fibrosis, lung, epithelium, *Pseudomonas aeruginosa*, lncRNA

## Abstract

*Pseudomonas aeruginosa* (*Pa*) is the leading cause of chronic lung infection in Cystic Fibrosis (CF) patients. It is well recognized that CF epithelial cells fail to develop an appropriate response to infection, allowing bacterial colonization and a chronic inflammatory response. Since long non-coding RNAs (lncRNAs), are known to play a key role in regulating mammalian innate immune response, we hypothesized that CF cells exposed to *Pa* could express a specific lncRNA signature responsible of the maladaptative CF response. We analyzed transcriptomic datasets to compare the expression profiles of lncRNAs in primary CF and non-CF epithelial cells infected with *Pa* at 0, 2, 4, and 6 h of infection. Our analysis identified temporal expression signatures of 25, 73, 15, and 26 lncRNA transcripts differentially expressed at 0, 2, 4, and 6 h post-infection respectively, between CF and non-CF cells. In addition, we identified profiles specific to CF and non-CF cells. The differential expression of two candidate lncRNAs were independently validated using real-time PCR. We identified a specific CF signature of lncRNA expression in a context of *Pa* infection that could potentially play a role in the maladaptive immune response of CF patients.

## Introduction

Cystic fibrosis (CF) is an autosomal recessive genetic disorder affecting one in 3,500 live births, in Caucasian populations. This disease is due to mutations in the cystic fibrosis transmembrane conductance regulator (*CFTR*) gene encoding the transporter channel for chloride and bicarbonate ions in epithelial cells. In airways, a deficient CFTR results in excessive secretion of abnormally thick and viscous mucus with impairment of innate immune host defenses. This results in chronic infection and inflammation, which together acts to further deteriorate the lung function (Palmer and Whiteley, 2015). *Pseudomonas aeruginosa* (*Pa*) is the leading cause of chronic lung infections and is responsible for significant morbidity and mortality in CF patients. Chronic *Pa* infection is characterized by repeated episodes of pulmonary exacerbations, during which lung function declines abruptly (Elborn, 2016).

Innate immunity system provides immediate defense against microbial invasion by recruiting immune cells and driving inflammation signals to sites of infection. Response of pulmonary epithelial cells to external stimuli requires tight regulation of genes involved in the innate immune response. This fine-tuned regulation, at the transcriptional and post-transcriptional levels, is necessary to prevent detrimental effects of uncontrolled cell activation. In a previous study, we compared transcriptomes of CF and non-CF bronchial epithelial cells during *Pa* infection (Balloy et al., 2015). We observed different profiles with genes up- and down-regulated in CF compared to non-CF cells that could illuminate the inflammatory response or be involved in the fighting of pathogens as well as in the decreased protection of tissue integrity.

Non-coding RNAs (ncRNAs) have recently emerged as important regulators of gene expression. Long ncRNAs (lncRNAs) larger than 200 nucleotides, represent the largest class of the ncRNA molecules. Although the current strongest evidence supports their role in cancer (Tsai et al., 2011), lncRNAs play key roles in regulating mammalian innate immune responses as well (Sigdel et al., 2015). Depending on their localization, they act on gene expression through interactions with DNA, RNA or proteins (Rinn and Chang, 2012) through various mechanisms including chromatin remodeling, epigenetic regulation, transcription, mRNA splicing, RNA decay and enhancer functions (Li et al., 2016).

It has been previously shown that lncRNA expression profile is altered in inflammatory lung diseases such as chronic obstructive pulmonary diseases (COPD), asthma and acute lung injury (ALI) (Xie and Liu, 2015). In CF, the description and the role of lncRNAs is beginning to be unraveled. However, *CFTR* expression and chloride ion function was recently shown to be regulated by lncRNA (McKiernan et al., 2014). A total of 1,063 lncRNAs with differential expression were identified from CF bronchial brush sampling compared to non-CF (Saayman et al., 2016). In particular, XIST and TLR8-AS1 were confirmed to be respectively up- and down-regulated in CF compared to non-CF samples. However, in this study, the influence of infection was not addressed, although there are increased evidences of lncRNA involvement in infectious diseases (Scaria and Pasha, 2012). Reports have revealed roles for lncRNAs as regulators of antimicrobial functions such as the lncRNA NEAT1 which directly regulates HIV replication (Zhang et al., 2013) or the lncRNA NRAV that functions as a negative regulator of the host antiviral immunity by repressing the expression of IFN-stimulated genes through strict control of the transcription rate (Ouyang et al., 2014).

We therefore investigated the differential expression of lncRNAs in CF and non-CF epithelial cells during infection by *Pa* that may partly mimic and possibly explain the differential mRNA profiles between the two cell types observed previously (Balloy et al., 2015). In the present study, we re-analyzed the RNA-seq data with this perspective and evaluated the expression profiles of lncRNAs in infected CF and non-CF epithelial cells at the aim of identifying a specific CF signature for lncRNA expression in the presence of *Pa* infection.

## Methods

### Dataset

Raw datasets publicly available (European Nucleotide Archive (ENA), primary accession number PRJEB9292) were from a transcriptional analysis designed to compare the molecular determinants of CF and non-CF epithelial cells to *Pa* infection. The PAK strain of *Pa* (given by Stephen Lowry) is relevant for pulmonary infections in the context of CF Like most of clinical strains responsible for CF infection, it expresses a full complement of virulence factors, including pili, flagella, type II secreted enzymes, type III secreted exo-enzymes S, T, and Y, exotoxin A, elastase and phospholipase. The data were generated from human bronchial epithelial cells from four CF patients homozygous for the p.F508del mutation and four healthy donors (non-CF), each infected with *Pa* at 0.25 Multiplicity of Infection (MOI) for 0, 2, 4, and 6 h. The data with an average of over 150 million reads with each data set were previously published (Balloy et al., 2015).

### Reads alignment, mapping, and assembly

Read quality was checked with FastQC program, a quality control tool for high throughput sequence data (http://www.bioinformatics.babraham.ac.uk/projects/fastqc/) and reads were further aligned to the hg38 build of the human genome (UCSC Genome Browser). The splice-aware aligner Tophat (Mortazavi et al., 2008) (v2.0.11) which is integrated with Bowtie2, a fast and sensitive gapped read aligner, was used for aligning the sequence reads. One dataset was excluded from further analysis after observing only less than 85% of read alignment. Datasets, raw reads and the proportion of aligned reads are summarized in Supplementary Table 1. The expression levels across the transcripts and genes were based on GENCODE v23 (http://www.GENCODEgenes.org/). Briefly, this annotation set comprised of a total of 198,619 transcripts in the human genome, including 79,795 protein-coding and 118,824 non-coding transcripts, among which 27,817 lncRNAs. The expression levels were computed as fragments per kilobase of exon per million (FPKM) units and the differential expression between the different sets were computed using *cuffdiff*.

### Identifying potential differentially expressed lncRNA transcripts

We used a nominal cutoff of 1 FPKM to filter genes and transcripts. The Supplementary Table 2 lists out the number of genes and transcripts covered by each sample within the range of FPKM ≥ 1, 5, and 10. The differentially expressed genes and transcripts were filtered using stringent cutoffs. We considered lncRNAs with FPKM ≥ 1 with a log2-fold change ≤ −1 or ≥1 and *p* ≤ 0.05 as differentially expressed.

We performed two orthologous analyses across datasets. We first determined genes and transcripts which were differentially up- or down-regulated at each infection time points between CF and non-CF epithelial cells. Then, we examined the lncRNA transcripts differentially expressed between the 0 h time point and 2, 4, and 6 h post-infection in both CF and non-CF groups analyzed separately. From this, we sorted out the differentially expressed lncRNA transcripts which were found only in CF or in non-CF cells. The overlapped transcripts of the first set comprising the differentially expressed lncRNA transcripts of CF/non-CF analysis and second set comprising the differentially expressed transcripts at 2, 4, and 6 h post-infection, identified only in CF cells, and gave us the potential lncRNA transcripts with potential for association with *Pa* infection in CF epithelial cells.

### Validation of expression level using qRT-PCR

Quantitative PCR was performed using an ABI StepOnePlus™ (Applied Biosystems, Carlsbad, CA, USA) and TaqMan technology. TaqMan probes used (Applied Biosystems) were XIST (Hs01079824), MEG9 (Hs01593046), BLACAT1 (Hs03839366), and GAPDH (Hs02758991). Relative quantifications of the lncRNA level were carried out using the 2^−ΔΔCt^ method, and normalized with respect to *GAPDH* and for the expression levels to respective non-infected cells (T0). Each sample was assessed in triplicates to ensure experiment quality.

### Statistical analysis

Data were described as mean ± SEM. Between-group differences were tested using the paired *t*-test. Values of p lower than 0.05 were considered significant; in the figures, statistically significant differences with *p* < 0.05 (^*^), *p* < 0.01 (^**^), and *p* < 0.001 (^***^) are indicated.

### Correlation analysis of gene expression data

Correlation analysis was carried out using Pearson's algorithm with the help of R package; rcorr. We observed the mRNA transcripts positively or negatively correlated with the overlapped lncRNAs from two analyses. Transcripts with an empirical r-score greater than 0.85 were considered to be positively regulated whereas transcripts with r-score less than −0.85 were considered to be negatively regulated and *p*-value less than 0.01 was considered as significant. We additionally performed enrichment analysis for the protein coding genes co-regulated with the lncRNAs using DAVID functional annotation tool.

## Results

### Identification of temporal infection signatures from RNA-seq data

**Figure 1:**
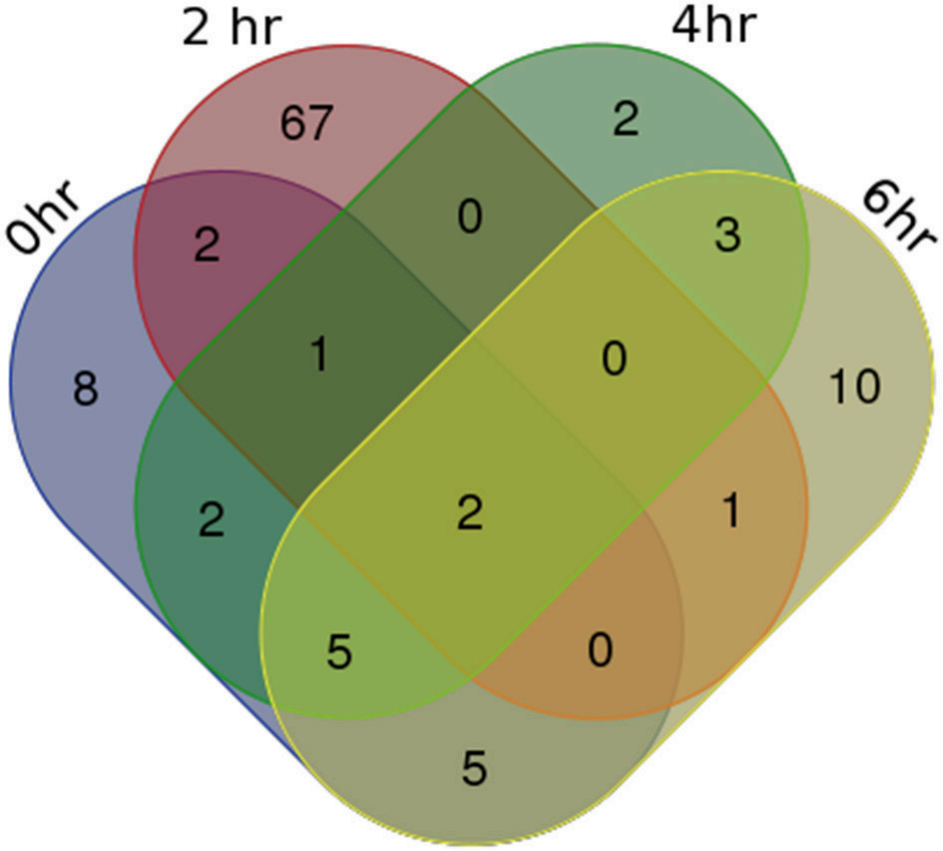
**Venn Diagram of differentially expressed lncRNA transcripts in CF vs. non-CF cells at each time point of infection**.

For identifying differentially expressed genes and transcripts in CF compared to non-CF epithelial cells, we used *cuffdiff* and the reference gene and transcript annotations from the GENCODE version 23 for guiding the assembly. The *cuffdiff* output provided FPKM values, log2-fold change value of CF upon non-CF for every time points studied. The numbers of genes differentially expressed at 0, 2, 4, and 6 h are found to be 297, 958, 281, and 439 respectively and a total of 336, 958, 336, and 518 transcripts respectively (Table 1). Their FPKM values and fold changes are summarized in Supplementary Table 3. The lncRNA annotation files were separately retrieved from the GENCODE database to extract all the significantly differentially expressed lncRNA transcripts. We identified a total of 25, 73, 15, and 26 differentially expressed lncRNAs, corresponding to the 0, 2, 4, and 6 h time points. The unique number of lncRNA transcripts differentially expressed throughout the dataset was found to be 108. These latter are shown in a Venn diagram (Figure 1) to visualize shared lncRNAs differentially expressed between CF and non-CF infected cells. Out of the 108 lncRNA transcripts, a total of 12 lncRNAs are differentially expressed in at least 2 time points. Table 2 lists these lncRNAs, their cognate gene locus and functions as annotated from literature. Three of the 12 lncRNAs have been previously shown to be expressed in the human lung. The lncRNA RP11-44F21.5 has been previously suggested to be down-regulated in squamous cell carcinoma of the lung in the following analysis: https://genevisible.com/perturbations/HS/Gene%20Symbol/RP11-44F21.5. The two other lncRNAs, LINC00704 and LINC00992, have been shown to be expressed in lungs, kidneys and salivary glands (Stelzer et al., 2016).

**Table 1:** **Summary of genes and transcripts differentially expressed in CF vs. non-CF cells at each time point of infection**.

**Differentially expressed genes/transcripts**	**0 h (CF/non-CF)**	**2 h (CF/non-CF)**	**4 h (CF/non-CF)**	**6 h (CF/non-CF)**
Genes	297	958	281	439
Transcripts	336	958	336	518
lncRNA transcripts	25	73	15	26
lncRNA genes	21	73	15	22
Unique lncRNA transcripts	108			
Unique lncRNA genes	104			

**Table 2:** **Summary of lncRNA genes differentially expressed in CF vs. non-CF cells for at least 2 of the time points of infection**.

**Conditions differentially expressed**				**lncRNA**	**Ensembl ID**	**Function**
**0**	**2**	**4**	**6**			
				CTD-2619J13.13	ENST00000596379.1 ENSG00000268307.1	CTD-2619J13.13 is a processed transcript type of long coding RNA. It belongs to chromosome 19 on the forward strand comprising 459 bp.
				LINC00862	ENST00000367356.1 ENSG00000203721.5	LINC00862 is a long Intergenic Non-Protein Coding RNA 862. It is also known as Small Integral Membrane Protein 16 or C1orf98. It belongs to chromosome 12, comprising 31,811 bp on reverse strand.
				CMB9-55F22.1	ENST00000623846.1 ENSG00000279672.1	CMB9-55F22.1 is a known pseudogene which is annotated as long coding RNA. It is found to have expressed in acute monoblastic/monocytic leukemia cell lines. It belongs to chromosome 11, encompassing 1,139 bp on forward strand
				POLR2J4	ENST00000427076.5 ENSG00000214783.9	POLR2J4 is expanded as Polymerase (RNA) II (DNA Directed) Polypeptide J4. It is a pseudogene, which is annotated as long coding RNA. It is also known as RPB11-Phi. It is found to be on chromosome 12 carrying 78,300 bps on reverse strand. It is studied to be expressed in majorly all tissue types.
				WFDC21P	ENST00000566140.5 ENSG00000261040.6	WFDC21P is expanded as WAP Four-Disulfide Core Domain 21 which is a pseudogene type of long coding RNA. It is also known as lncDC. The STAT3-Binding Long Non-coding RNA lnc-DC Controls Human Dendritic Cell Differentiation. It belongs to chromosome 17 containing 8,320 bp on reverse strand
				RP11-442H21.2	ENST00000491934.2 ENSG00000269926.1	RP11-442H21.2 is a known antisense long non-coding RNA. It is found to be expressed in bone marrow and cord blood. It belongs ro chromosome 10 comprising 1,066 bp on reverse strand.
				RP11-890B15.2	ENST00000533812.6 ENSG00000254842.6	RP11-890B15.2 is a known long intergenic non-coding RNA. It has found to have some regulatory activities like CTCF binding, promoter seq binding. It belongs to chromosome 11 comprising 2,203 bps on reverse strand.
				RP11-445P17.8	ENST00000449457.1 ENSG00000224034.1	RP11-445P17.8 is a known lincRNA. It has found to be expressed in gallbladder and acting as an enhancer. It belongs to chromosome 10, 5,204 bp long on reverse strand.
				RP11-44F21.5	ENST00000561705.1 ENSG00000260265.1	RP11-44F21.5 is a known lincRNA. It has found to be expressed in bladder and brain. The lncRNA is studied to be downregulated in pancreatic cancer studies and lung squamous carcinoma with normal non-cancerous tissues while found upregulated between allergic house dust mite and non-allergic dust mite. This belongs to chromosome 4 comprising 3,016 bps.
				RP11-495P10.8	ENST00000434245.2 ENSG00000231196.3	RP11-495P10.8 is a long intergenic non coding RNA which has shown role in epigenetic modifications in liver and brain cells. The overall mean expression of RP11-495P10.8 from different datasets is 0.20 RPM, ranging from as low as 0.00 RPM to 7.29 RPM. It is studied to be expressed more in erythrocytic leukemia. It belongs to chromosome 1 spanning 4,971 bp on reverse strand.
				LINC00704	ENST00000430998.6 ENSG00000231298.6	LINC00704 is an RNA Gene, and is affiliated with the non-coding RNA class. It is found to have expressed in internal organs like lungs and kidneys and secretory tissue like salivary and pituitary gland. It belongs to chromosome 10 comprising 27,970 bps.
				LINC00992	ENST00000504107.1 ENSG00000248663.6	LINC00992 expanded as Long Intergenic Non-Protein Coding RNA 992 is an RNA Gene, and is affiliated with the non-coding RNA class. It is also known as CTC-504A5.1. It is studied to have expressed in internal organs like lungs and kidneys and secretory tissue like salivary and pituitary gland. It belongs to chromosome 5 spanning the size 164,236 bps on forward strand.

### Highlighting distinct specific infection signatures in non-CF and CF cells

**Figure 2:**
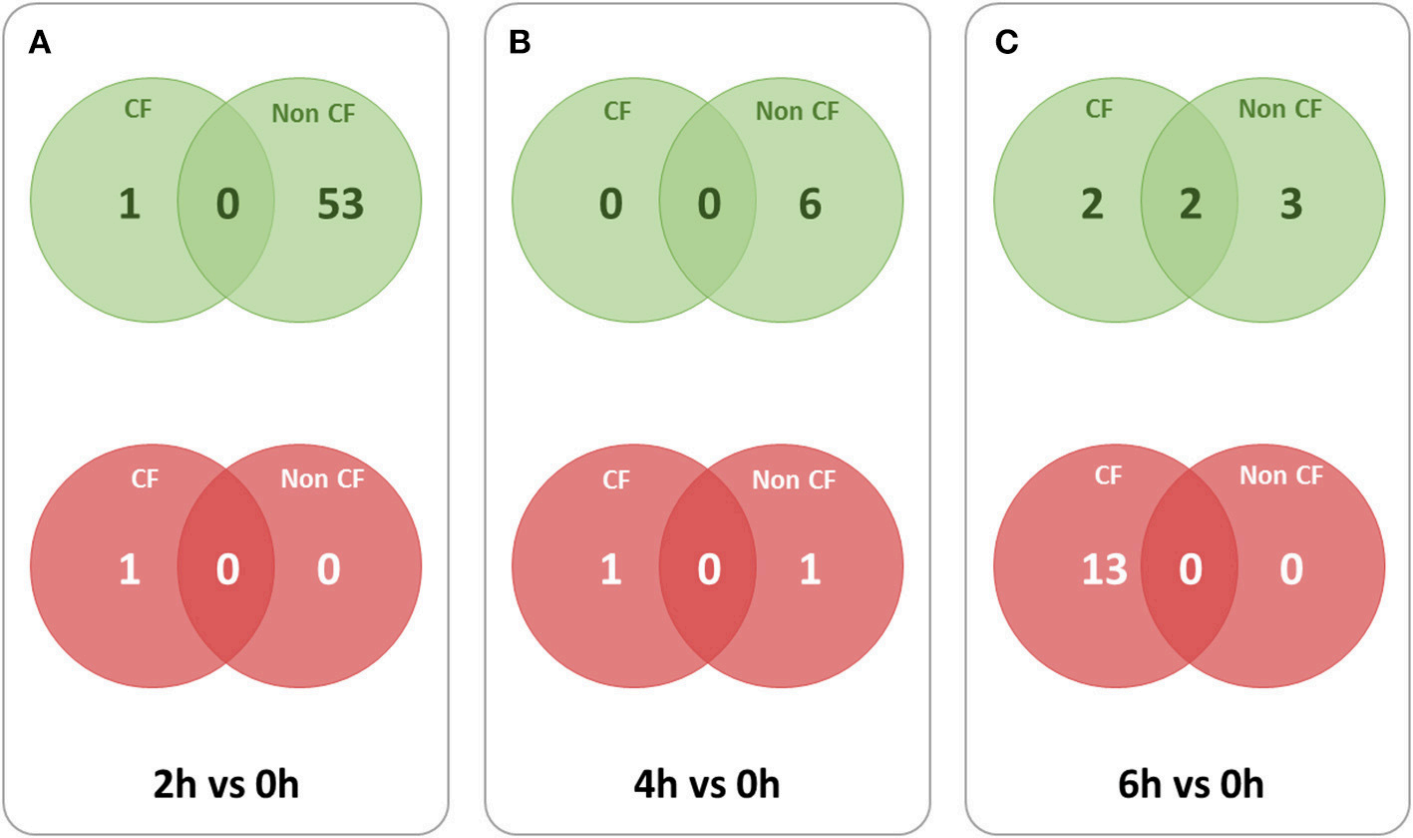
**Overlaps between lncRNA transcripts differentially expressed at different time points in CF and non-CF datasets**. Red circles represent down-regulated transcripts and green circles represent up-regulated transcripts. **(A)** 2 h in comparison with 0 h time point, **(B)** 4 h in comparison with 0 h time point, **(C)** 6 h in comparison with 0 h time point.

To identify the lncRNA transcripts specific of *Pa* infection, we used *cuffdiff* to compare the expression values for each time point against 0 h for CF and non-CF datasets separately. We retrieved the differentially expressed transcripts using the same filters as mentioned previously. The total number of genes or transcripts, differentially expressed were found comparatively less in CF than in non-CF samples at 2 h vs. 0 h (Table 3). We further analyzed the common lncRNA transcripts differentially expressed at each time point in CF and non-CF datasets. Except for 2 up-regulated lncRNAs at 6 h vs. 0 h, we obtained two distinct profiles of lncRNAs as none of lncRNAs differentially expressed during infection in non-CF are common to those differentially expressed in CF cells (Figure 2). The FPKM values and fold changes of the up- and down-regulated transcripts are summarized in Supplementary Tables 4, 5, respectively.

**Table 3:** **Genes and lncRNAs differentially expressed in CF and non-CF cells at different time points of infection compared to the non-infected status (0 h)**.

**Differentially expressed genes/transcripts**	**2 h vs. 0 h**		**4 h vs. 0 h**		**6 h vs. 0 h**	
	**CF**	**Non-CF**	**CF**	**Non-CF**	**CF**	**Non-CF**
Genes	72	587	99	75	272	140
Transcripts	72	603	102	76	305	146
lncRNA transcripts	2	53	1	7	17	5
lncRNA Genes	2	53	1	7	17	5
Unique lncRNA transcripts	80					
Unique lncRNA genes	79					

**Figure 3:**
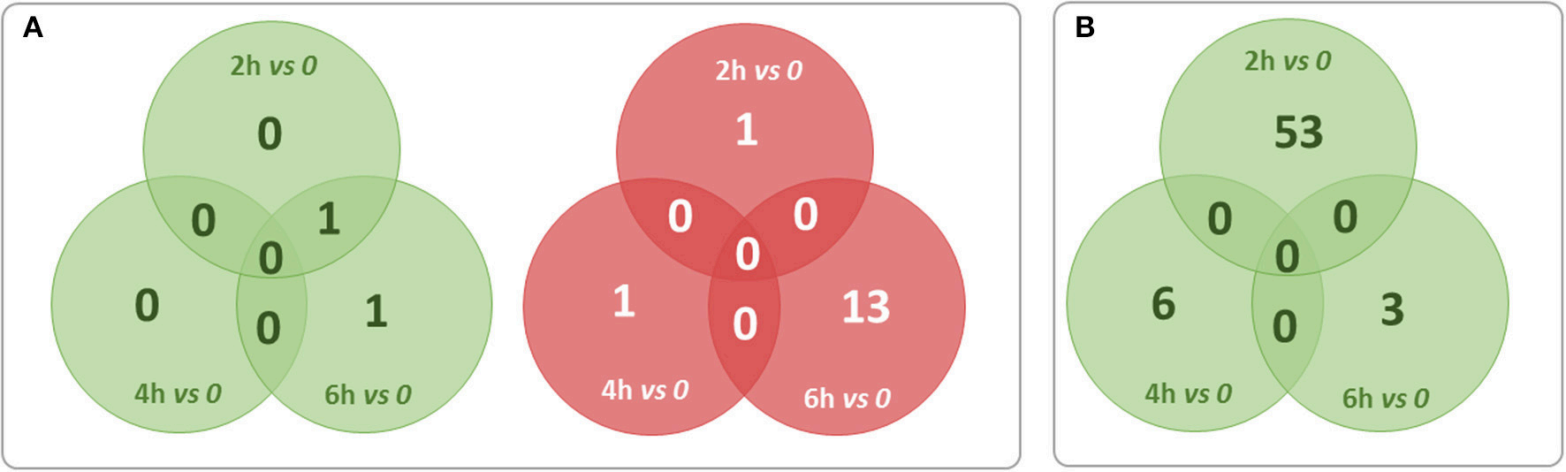
**Summary of overlaps between differentially expressed transcripts only in the CF set (A)** and only in non-CF set **(B)** at each of the respective time points against non-infected time point (0 h). Red circles represent down-regulated transcripts and green circles represent up-regulated transcripts. No transcripts were found down-regulated exclusively in non-CF set.

For finding out the lncRNA signature specific to infected CF cells in the one hand and to non-CF cells in the other hand, we summarized overlaps between differentially expressed (both up and down) transcripts in the CF sets (Figure 3A) and in the non-CF sets (Figure 3B) respectively for each of the respective time points against non-infected time point (0 h). We found that 62 lncRNA transcripts (62 up-regulated) are specifically regulated in *Pa* infected non-CF epithelial cells, whereas 17 lncRNA transcripts (2 up- and 15 down-regulated) are specific of the CF epithelial cell response to the infection. The FPKM values and fold changes of the up-regulated transcripts in non-CF cells and the up- and down-regulated in CF cells are summarized in Supplementary Tables 6–8, respectively.

**Figure 4:**
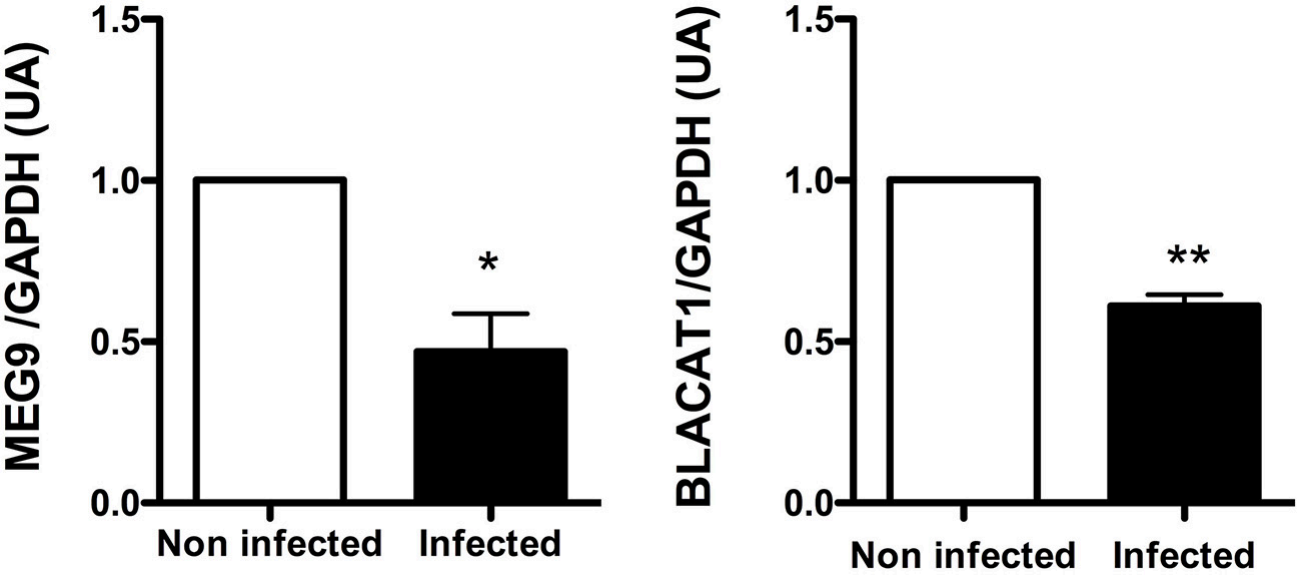
**Validation by qRT-PCR of BLACAT1 and MEG9 expressions in bronchial primary epithelial CF cells infected with *Pa***. RNA were purified in CF cells collected from 4 different patients and infected with *Pa*. LncRNA levels were measured at 0 (white bar) and 2 h (black bar) post-infection for MEG9 and 0 (white bar) and 6 h (black bar) post-infection for BLACAT1. The statistical analysis consisted in a paired *t-*test. ^*^P < 0.05 and ^**^P < 0.001.

Among the lncRNA transcripts specific signature of infected CF cells, we selected two of them already studied in the literature, MEG9 and BLACAT1, for validation of their expression by qPCR. Thus, the pattern of expression found by *in silico* analysis for MEG9 and BLACAT1 was confirmed with a significant down regulation at 2 and 6 h post-infection, respectively (Figure 4). One lncRNA transcript, XIST, identified in the infected non-CF signature and already described by Greene's team comparing CF and non-CF samples, was measured by qPCR. Validation of its expression was not as clear as for MEG9 and BLACAT1. Indeed, we observed varying levels of expression between epithelial cells derived from male and female, independently of CFTR genotype, i.e., very low or no expression in male cells (Ct around 36 or undetermined) whereas high expression in female cells (Ct around 18.5) (data not shown).

### Proteins coding transcripts correlated with BLACAT1 and MEG9 expression

The Gene Ontology analysis did not reveal protein-coding genes significantly correlated with BLACAT1 expression, but it identified protein coding transcripts positively and negatively correlated with lncRNA MEG9. Among the genes positively correlated, we observed 62.3% of proteins engaged in protein binding, 18.5% in positive regulation of cellular process and around 10% in extracellular matrix. The genes negatively correlated (17.4%) with lncRNA MEG9 were involved in structural components of ribosome (Table 4).

**Table 4:** **DAVID's GO terms for protein coding transcripts positively and negatively correlated with the lncRNA MEG9**.

**Category**	**GO term**	**Count**	**Percentage**	***P*-value**	**Benjamini corrected *p*-value**
**GENES POSITIVELY CORRELATED IN EXPRESSION**					
Biological process	Regulation of localization	19	9.2	3.0E-4	4.6E-2
	Positive regulation of cellular process	38	18.5	7.0E-4	5.30E-02
Cellular components	Extracellular matrix part	10	4.8	1.3E-5	4.4E-4
	Extracellular matrix	14	6.8	3.1E-4	5.3E-3
Molecular function	Protein binding	129	62.3	1.60E-4	7.80E-3
**GENES NEGATIVELY CORRELATED IN EXPRESSION**					
Molecular function	Structural constituent of ribosome	4	17.4	1.9E-3	3.10E-2

## Discussion

The airway epithelium is the first site in contact with inhaled pathogens. Its response to bacterial invasions activates innate immune mechanisms through the involvement of various receptors. In CF patients, this normal process is disturbed leading to a decrease of the bacterial clearance resulting in chronic infection in lungs.

Over the years, lncRNAs have been recognized as major regulators of multiple cellular processes among which the regulation of immune effectors. They can interact with transcription factors which is the case of the lncRNA NRON that inhibits the translocation of NFAT in the nucleus by sequestering it in the cytosol and so negatively regulating T-cell activation (Willingham et al., 2005). Three lncRNAs (murine NeST, human THRIL, and NEAT1) have been previously shown to regulate the innate immune response by modulating the transcription of cytokines such as IFN-γ, TNF-α, and IL-8 (Cullen, 2013; Gomez et al., 2013; Imamura et al., 2014; Li et al., 2014). It is also known that toll-like receptor (TLR) signaling can activate endogenous feedback-regulation networks to limit the potentially damaging effects of an excessive inflammation. This is the case with the lncRNA THRIL which helps in down-regulation to restrain TLR-induced gene activation (Li et al., 2014).

LncRNA expression is tissue-specific indicating a tight regulation that could be affected by *Pa* infection in CF patients. For this reason, we decided to explore the expression of lncRNAs in the infectious context of respiratory epithelial cells that are key cells involved in CF. CF vs. non-CF infected cells study brought 108 unique lncRNAs as differentially up- or down-regulated with 12 lncRNAs which are expressed in at least two time points. Among them, the lncRNAs, LINC00862, and CTD-2619J13, are differentially expressed at all four time points in spite of bacterial invasion. As such, they could be two potential signatures for CF. Most of the 108 unique lncRNAs was differentially expressed at 2 h, almost immediately after bacterial attack. According to Ensembl and lncRNAdb, some of these differentially expressed lncRNAs are involved in regulatory activities namely CTCF binding, promoter sequence binding transcripts and few were found differentially expressed in different tissues such as in lungs and in erythrocytes.

To pinpoint a specific signature related to infection in the CF vs. non-CF context, we took in account lncRNA transcripts specific to *Pa* infection expressed only in CF in one hand and in non-CF cells in other hand from each time point (i.e., 2, 4, and 6 h) and compared with their expression in uninfected cells.

Numbers of lncRNAs differentially expressed for CF and non-CF cells are totally different with 17 and 62 lncRNAs, respectively. Among the lncRNAs belonging to the specific signature of *Pa* infected non-CF cells; we observed a well annotated lncRNA, XIST. As described by Greene's team ^9^, we detected the lncRNA XIST more expressed in CF than in non-CF cells at the non-infected status (Fold change >4). Moreover, we observed that XIST expression is up-regulated in non-CF cells during infection at 4 and 6 h (FC > 2). Nonetheless, the validation of XIST by qPCR does not fit with the *in silico* analysis. Indeed, we observed differences of expression between cells from male and female donors but no differences of expression between CF and non-CF epithelial cells. This result is consistent with the literature as XIST is a lncRNA required for the establishment of X-chromosome silencing in placental mammals. X inactivation is an early developmental process in mammalian females that transcriptionally silences one of the pair of X chromosomes, thus providing dosage equivalence between males and females (Furlan and Rougeulle, 2016). Due to the differences in the gender of donors, taken individually, values did not show the variations averaged *in silico* analysis. Other transcripts, ENST00000452120.6 and ENST00000398461.5 were found in the CF cells specific signatures. These transcripts are annotated as MEG3 (Maternally Expressed 3) which is a maternally imprinted gene with all spliced transcripts annotated as lncRNAs and is a well-known lncRNA tumor suppressor. MEG3 has been previously reported to regulate the TGF-β pathway genes by forming RNA-DNA triplex structures (Mondal et al., 2015). This pathway is frequently regulated during tissue injury and repair and the up-regulation of TGF-β is involved in lung fibrogenic diseases. The expression of the lncRNA MEG3 in signature could be associated with the absence of injury after invasion of *Pa* in non-*CF*. A recent study in mice characterized MEG3 as a novel pulmonary inflammatory regulator of bacterial infection through miR-138 (Li and Wu, 2016).

The highlighting of a specific signature in CF epithelial cells in response to *Pa* infection revealed 17 lncRNA transcripts. Among them, 4 lncRNA transcripts MEG9, BLACAT1, RP11-477I4.4, and RP11-1334A24.5 overlapped with the 108 unique lncRNAs which are differentially expressed between CF and non-CF cells. RT-PCR gave a similar pattern of expression of MEG9 and BLACAT1 as from *in silico* analysis and validate the different signatures obtained with the computational analysis. ENST00000626538.1, otherwise known as lncRNA BLACAT1 (Bladder Cancer Associated Transcript 1), was found down-regulated in CF patients. Until now, BLACAT1, firstly characterized in bladder cancer, was also described to be involved in the development of gastric cancer. It has been shown that its depletion decreased the gastric cancer cell proliferation, motility and invasion (Hu et al., 2015). Since shedding and abnormal repair are characteristics of CF epithelial cells, it would be interesting to study whether their altered expression of BLACAT1 influences their repair, proliferation and migration. In our study, analysis did not reveal protein-coding gene significantly correlated with BLACAT1 expression. However, as shown previously BLACAT1 could interact directly with other molecular partners than transcripts such as the proteins of the polycomb repressive complex 2 (PRC2) (He et al., 2013).

Concerning MEG9 (Maternally Expressed 9), a lncRNA belonging to the MEG family, it has been described to be up-regulated upon hypoxia in human endothelial cells and in a model of mouse ischemia (Voellenkle et al., 2016). In our study, MEG9 was found dramatically down-regulated 2 h post-infection compared to the non-infected status (0 h) in CF cells (Supplementary Table 8). From our correlation analysis of gene expression, MEG9 was found co-regulated positively with 226 and negatively with 25 protein coding genes. Gene ontology analysis showed that MEG9 was found positively co-regulated mostly with proteins engaged in protein binding (62.3%), in positive regulation of cellular process (18.5%) and few of them in extracellular matrix (Table 4), all processes involved in inflammation of the lung in CF (Cohen-Cymberknoh et al., 2013).

Our study showed that *Pa* infection altered the expression of lncRNAs in CF respiratory epithelial cells which could have a potential role in the innate immune system and thus could contribute to the maladaptive immune response observed in patients. We have experimentally validated the two potential transcripts namely MEG9 and BLACAT1 expression, which were found differentially expressed in both CF/non-CF and infected/non-infected analyses. Further investigations either *in vitro* (respiratory epithelial cells in culture) or *in vivo* (study of conserved LncRNA (MEGs) in mice model of pulmonary infection) are necessary to understand the role of these lncRNAs in the innate immune response. The present study may contribute to provide further insight into the biological functions and molecular mechanisms of lncRNAs regulation in CF in which *Pa* infection plays a crucial role.

## Author contributions

Conceived and designed the experiments: LG, VB, and MC. Performed the biological experiments: LG, VB. Analyzed the data: RK, LG, VB, and VS. Wrote the paper: RK, VB, LG, MC, LP, HC, and VS.

### Conflict of interest statement

The authors declare that the research was conducted in the absence of any commercial or financial relationships that could be construed as a potential conflict of interest.
