# Supplementary material for: Bronchial Epithelial Cells from Cystic Fibrosis Patients Express a Specific Long Non-coding RNA Signature upon Pseudomonas aeruginosa Infection
Source: Front Cell Infect Microbiol. 2017 May 29;7:218. doi: 10.3389/fcimb.2017.00218 (PMC5447040; doi:10.3389/fcimb.2017.00218)
Supplement: Supplementary file 1 [file Table1.PDF]

Supplementary Table 1. Summary of the datasets, samples and descriptions, reads sequenced in each dataset and mapping rate across the human reference genome.

Sl No	Sample ID	Description	Raw Reads	Filtered Reads	Mapped Reads	Mapping rate
1	VLM_1	Control cell at 0 h (N1)	175,905,909	175,905,909	165,082,967	93.8
2	VLM_2	Control cell at 0 h (N2)	189,466,711	189,466,711	178,842,113	94.4
3	VLM_3	Control cell at 0 h (N3)	176,138,649	176,138,649	164,941,819	93.6
4	VLM_4	Control cell at 0 h (N4)	179,739,079	179,739,079	169,460,894	94.3
5	VLM_5	Cystic fibrosis cell at 0 h (CF1)	186,944,216	186,944,216	177,352,492	94.9
6	VLM_6	Cystic fibrosis cell at 0 h (CF2)	172,919,572	172,919,572	162,870,006	94.2
7	VLM_7	Cystic fibrosis cell at 0 h (CF3)	151,208,855	151,208,855	141,215,818	93.4
8	VLM_8	Cystic fibrosis cell at 0 h (CF4)	185,424,259	185,424,259	174,299,098	94
9	VLM_9	Control cell at 2 h (N1-2h)	189,856,427	189,856,427	179,779,647	94.7
10	VLM_10	Control cell at 2 h (N2-2h)	177,609,745	177,609,745	169,211,291	95.3
11	VLM_11	Control cell at 2 h (N3-2h)	156,956,338	156,956,338	146,133,284	93.1
12	VLM_12	Control cell at 2 h (N4-2h)	115,521,167	115,521,167	100,830,961	87.3
13	VLM_13	Cystic fibrosis cell at 2 h (CF1-2h)	192,395,205	192,395,205	181,027,724	94.1
14	VLM_14	Cystic fibrosis cell at 2 h (CF2-2h)	166,786,183	166,786,183	157,304,190	94.3
15	VLM_15	Cystic fibrosis cell at 2 h (CF3-2h)	71,776,749	71,776,749	62,279,066	86.8
16	VLM_16	Cystic fibrosis cell at 2 h (CF4-2h)	169,615,009	169,615,009	156,946,530	92.5
17	VLM_17	Control cell at 4 h (N1-4h)	173,892,419	173,892,419	163,545,613	94
18	VLM_18	Control cell at 4 h (N2-4h)	188,825,462	188,825,462	177,908,538	94.2
19	VLM_19	Control cell at 4 h (N3-4h)	181,957,481	181,957,481	167,158,784	91.9
20	VLM_20	Control cell at 4 h (N4-4h)	178,852,479	178,852,479	169,470,373	94.8
21	VLM_21	Cystic fibrosis cell at 4 h (CF1-4h)	177,843,459	177,843,459	167,147,902	94
22	VLM_22	Cystic fibrosis cell at 4 h (CF2-4h)	152,978,910	152,978,910	142,686,981	93.3
23	VLM_23	Cystic fibrosis cell at 4 h (CF3-4h)	167,733,140	167,733,140	157,642,057	94
24	VLM_24	Cystic fibrosis cell at 4 h (CF4-4h)	128,354,705	128,354,705	113,846,788	88.7
25	VLM_25	Control cell at 6 h (N1-6h)	185,463,754	185,463,754	176,095,655	94.9
26	VLM_26	Control cell at 6 h (N2-6h)	132,114,167	132,114,167	120,926,581	91.5
27	VLM_27	Control cell at 6 h (N3-6h)	189,096,773	189,096,773	176,672,792	93.4
28	VLM_28	Control cell at 6 h (N4-6h)	51,955,720	51,955,720	46,543,351	89.6
29	VLM_29	Cystic fibrosis cell at 6 h (CF1-6h)	196,933,816	196,933,816	185,225,873	94.1
30	VLM_30	Cystic fibrosis cell at 6 h (CF2-6h)	129,531,918	129,531,918	117,169,951	90.5
31	VLM_31	Cystic fibrosis cell at 6 h (CF3-6h)	174,512,708	174,512,708	163,609,493	93.8
32	VLM_32	Cystic fibrosis cell at 6 h (CF4-6h)	180,043,794	180,043,794	118,341,443	65.7
