# Supplementary material for: Bronchial Epithelial Cells from Cystic Fibrosis Patients Express a Specific Long Non-coding RNA Signature upon Pseudomonas aeruginosa Infection
Source: Front Cell Infect Microbiol. 2017 May 29;7:218. doi: 10.3389/fcimb.2017.00218 (PMC5447040; doi:10.3389/fcimb.2017.00218)
Supplement: Supplementary file 2 [file Table2.PDF]

Supplementary Table 2: Gene and transcript counts covered by all samples in FPKM>=1,5 and 10 units

Sl No:	Dataset File	Genes covered FPKM =>1	Transcripts covered FPKM =>1	Genes covered FPKM =>5	Transcripts covered FPKM =>5	Genes covered FPKM =>10	Transcripts covered FPKM =>10
1	VLM_1	25665	29590	15902	16431	11985	11794
2	VLM_2	28391	32074	16097	16263	11395	11157
3	VLM_3	27030	30835	16123	16605	11944	11884
4	VLM_4	25971	29927	16293	16830	12197	12097
5	VLM_5	28086	32615	17092	17609	12651	12346
6	VLM_6	25428	29335	16145	16587	12034	11887
7	VLM_7	25909	29525	15374	15683	11281	11071
8	VLM_8	29152	33571	17515	17980	12813	12567
9	VLM_9	25292	28875	15404	15740	11378	11142
10	VLM_10	27368	31242	16185	16502	11827	11575
11	VLM_11	24000	26770	13027	13013	9139	8958
12	VLM_12	50743	52701	22776	23172	15555	15696
13	VLM_13	25741	29263	15206	15369	10951	10703
14	VLM_14	24402	27339	13695	13638	9605	9383
15	VLM_15	31699	36062	17661	18359	12793	12735
16	VLM_16	26677	30469	15972	16281	11666	11456
17	VLM_17	26123	30167	16437	16927	12330	12187
18	VLM_18	28450	32688	17246	17666	12628	12388
19	VLM_19	25509	29439	16037	16477	11824	11702
20	VLM_20	27568	31871	16905	17415	12382	12180
21	VLM_21	25753	29691	16102	16521	11941	11791
22	VLM_22	25623	29589	16237	16840	12258	12177
23	VLM_23	28007	32265	16988	17462	12469	12228
24	VLM_24	26609	30636	16141	16675	11928	11848
25	VLM_25	26553	29988	15270	15311	10698	10453
26	VLM_26	28198	32284	16910	17525	12504	12372
27	VLM_27	30838	27258	15539	15686	11040	10778
28	VLM_28	33444	38094	18524	19376	13477	13420
29	VLM_29	24566	26809	12296	12052	8211	8000
30	VLM_30	27524	31755	17071	17654	12773	12655
31	VLM_31	26705	30603	16518	16999	12207	12030
