# Supplementary material for: Bronchial Epithelial Cells from Cystic Fibrosis Patients Express a Specific Long Non-coding RNA Signature upon Pseudomonas aeruginosa Infection
Source: Front Cell Infect Microbiol. 2017 May 29;7:218. doi: 10.3389/fcimb.2017.00218 (PMC5447040; doi:10.3389/fcimb.2017.00218)
Supplement: Supplementary file 3 [file Table3.PDF]

Supplementary Table 3. Differentially expressed lncRNAs at different timepoints between CF and non-CF.

0h	FPKM		
Ensembl ID	CF	non-CF	FC
ENST00000367356.1	2.04	0.53	3.85
ENST00000398461.5	13.37	3.37	3.97
ENST00000429368.1	1.08	0.33	3.27
ENST00000429829.5	33.65	8.39	4.01
ENST00000430998.6	4.56	2.13	2.14
ENST00000449457.1	1.15	0.37	3.09
ENST00000452120.6	17.92	4.71	3.81
ENST00000487827.1	1.11	0.53	2.09
ENST00000491934.2	14.77	31.35	0.47
ENST00000498832.1	1.49	0.67	2.23
ENST00000524035.5	8.36	2.28	3.67
ENST00000531894.5	1.46	0.70	2.08
ENST00000533812.6	2.02	0.86	2.36
ENST00000548702.1	1.24	0.44	2.83
ENST00000553613.1	1.20	0.18	6.70
ENST00000560221.1	1.23	0.50	2.45
ENST00000561705.1	1.67	0.80	2.08
ENST00000566140.5	14.96	50.07	0.30
ENST00000574180.1	1.05	0.47	2.21
ENST00000587298.1	5.82	17.33	0.34
ENST00000589843.1	5.15	1.66	3.10
ENST00000592556.5	10.31	36.95	0.28
ENST00000596379.1	1.35	4.51	0.30
ENST00000618966.1	25.31	12.54	2.02
ENST00000624016.1	9.61	4.64	2.07

2hr	FPKM		
Ensembl ID	CF	non-CF	FC
ENST00000360656.2	0.66	3.17	0.21
ENST00000367356.1	1.94	0.55	3.50
ENST00000411579.1	2.01	6.41	0.31
ENST00000416221.5	13.95	46.90	0.30
ENST00000422971.1	0.20	1.07	0.18
ENST00000424948.1	0.26	1.03	0.26
ENST00000429829.5	20.62	4.26	4.84
ENST00000430998.6	3.77	1.63	2.31
ENST00000446211.1	0.27	1.54	0.17
ENST00000448587.5	1.54	3.14	0.49
ENST00000458733.5	0.57	6.88	0.08
ENST00000473970.2	0.98	4.23	0.23
ENST00000504107.1	0.57	1.39	0.41
ENST00000504573.1	0.30	1.20	0.25
ENST00000521399.5	97.39	303.98	0.32
ENST00000528887.1	2.69	66.73	0.04
ENST00000529934.5	3.78	38.00	0.10
ENST00000531523.1	23.21	146.25	0.16
ENST00000537269.1	25.83	77.49	0.33
ENST00000542022.1	7.46	131.79	0.06
ENST00000560221.1	1.12	0.38	2.93
ENST00000561761.2	0.43	1.03	0.42
ENST00000563192.1	44.50	147.25	0.30
ENST00000563605.1	4.16	152.76	0.03
ENST00000564240.1	0.44	1.99	0.22
ENST00000564248.1	2.55	11.97	0.21
ENST00000564650.1	0.90	2.00	0.45
ENST00000565152.1	0.35	1.96	0.18
ENST00000565382.1	0.56	2.15	0.26
ENST00000566876.1	0.90	5.01	0.18
ENST00000568280.1	1.08	2.50	0.43
ENST00000569087.2	1.50	4.34	0.35
ENST00000576215.1	1.56	95.46	0.02
ENST00000582866.1	1.12	3.01	0.37
ENST00000585496.1	13.72	303.03	0.05
ENST00000585742.1	4.30	69.57	0.06
ENST00000587088.1	9.23	270.36	0.03
ENST00000593554.1	0.41	2.10	0.20
ENST00000595428.1	0.52	2.99	0.18
ENST00000595563.1	0.13	11.05	0.01

4h	FPKM		
Ensembl ID	CF	non-CF	FC
ENST00000367356.1	2.05	0.61	3.39
ENST00000427076.5	0.72	1.64	0.44
ENST00000429368.1	1.10	0.38	2.89
ENST00000430998.6	6.75	2.96	2.28
ENST00000434245.2	0.08	1.13	0.07
ENST00000437686.1	0.42	2.74	0.15
ENST00000449457.1	1.07	0.51	2.09
ENST00000491934.2	19.18	55.44	0.35
ENST00000533812.6	1.79	0.76	2.37
ENST00000545177.4	1.06	0.16	6.58
ENST00000553613.1	1.44	0.45	3.22
ENST00000561705.1	1.71	0.59	2.88
ENST00000566140.5	16.71	34.01	0.49
ENST00000596379.1	1.55	4.16	0.37
ENST00000623846.1	0.67	1.87	0.36

6h	FPKM		
Ensembl ID	CF	non-CF	FC
ENST00000367356.1	1.60	0.59	2.74
ENST00000392688.6	0.46	1.63	0.28
ENST00000398461.5	12.91	3.87	3.34
ENST00000423943.1	9.92	22.61	0.44
ENST00000427076.5	0.47	1.21	0.39
ENST00000434245.2	0.08	1.62	0.05
ENST00000438324.1	0.28	1.29	0.22
ENST00000449457.1	1.08	0.41	2.62
ENST00000452120.6	19.03	6.11	3.11
ENST00000491934.2	31.12	92.09	0.34
ENST00000498832.1	1.86	0.68	2.75
ENST00000504107.1	0.57	1.21	0.47
ENST00000508179.1	2.65	1.30	2.04
ENST00000517927.1	3.37	1.15	2.92
ENST00000523411.1	0.12	1.02	0.11
ENST00000533812.6	1.51	0.71	2.13
ENST00000533992.1	1.11	0.49	2.25
ENST00000561705.1	2.12	0.52	4.06
ENST00000566140.5	14.99	44.74	0.34
ENST00000587298.1	5.34	15.80	0.34
ENST00000589987.5	6.22	19.50	0.32
ENST00000592556.5	10.93	30.29	0.36
ENST00000595748.1	0.40	1.92	0.21
ENST00000596379.1	1.90	7.36	0.26
ENST00000623846.1	0.57	1.41	0.40
ENST00000626538.1	0.55	1.16	0.48

2hr	FPKM		
**Ensembl ID**	**CF**	**non-CF**	**FC**
ENST00000596379.1	0.92	5.43	0.17
ENST00000599274.1	0.91	22.77	0.04
ENST00000602458.1	1.18	2.84	0.42
ENST00000602597.1	0.33	1.18	0.28
ENST00000602820.1	0.42	1.47	0.29
ENST00000602890.1	106.67	299.40	0.36
ENST00000606054.1	0.69	1.82	0.38
ENST00000606064.2	0.91	4.62	0.20
ENST00000606185.1	1.42	8.52	0.17
ENST00000606194.1	0.10	6.34	0.02
ENST00000607723.1	0.51	1.67	0.30
ENST00000607786.1	0.52	2.04	0.26
ENST00000607956.1	1.96	8.09	0.24
ENST00000608012.1	1.45	4.33	0.33
ENST00000609071.1	0.87	1.91	0.45
ENST00000609183.1	1.03	9.91	0.10
ENST00000609649.1	0.25	3.39	0.07
ENST00000610058.1	0.46	2.48	0.19
ENST00000610220.1	0.32	1.39	0.23
ENST00000610979.1	1.47	3.13	0.47
ENST00000612365.1	12.71	36.51	0.35
ENST00000613543.1	0.34	1.45	0.24
ENST00000614061.1	0.91	2.02	0.45
ENST00000614912.1	0.56	2.91	0.19
ENST00000616815.1	10.72	43.28	0.25
ENST00000617652.1	0.39	2.61	0.15
ENST00000622856.1	0.40	1.94	0.21
ENST00000623274.1	0.69	45.71	0.02
ENST00000623356.1	6.37	152.03	0.04
ENST00000623593.1	3.32	1.19	2.80
ENST00000624421.1	139.83	3241.04	0.04
ENST00000624988.1	1.60	11.02	0.14
ENST00000625139.1	0.78	5.52	0.14
