# Supplementary material for: Bronchial Epithelial Cells from Cystic Fibrosis Patients Express a Specific Long Non-coding RNA Signature upon Pseudomonas aeruginosa Infection
Source: Front Cell Infect Microbiol. 2017 May 29;7:218. doi: 10.3389/fcimb.2017.00218 (PMC5447040; doi:10.3389/fcimb.2017.00218)
Supplement: Supplementary file 4 [file Table4.PDF]

Supplementary Table 4. LncRNA transcripts Upregulated at 2h,4h,6h in comparison with 0h timepoints in CF and non CF groups

2h v/s 0h

CF	FPKM at 2h	FPKM at 0h	FC
ENST00000602361.1	209.60	33.17	6.32
**Non CF**	**FPKM at 2h**	**FPKM at 0h**	**FC**
ENST00000360656.2	3.17	0.44	7.13
ENST00000411579.1	6.42	2.09	3.07
ENST00000416221.5	46.99	17.80	2.64
ENST00000422971.1	1.07	0.28	3.80
ENST00000424948.1	1.03	0.23	4.52
ENST00000429630.1	24.56	10.72	2.29
ENST00000430034.1	35.39	1.91	18.50
ENST00000444464.1	1.25	0.33	3.76
ENST00000446211.1	1.54	0.28	5.50
ENST00000469903.5	30.14	8.38	3.59
ENST00000473970.2	4.22	1.01	4.19
ENST00000504573.1	1.20	0.22	5.37
ENST00000528887.1	66.54	2.97	22.42
ENST00000531523.1	145.95	22.69	6.43
ENST00000537269.1	77.50	18.73	4.14
ENST00000563192.1	147.23	39.77	3.70
ENST00000564248.1	11.98	2.99	4.01
ENST00000565152.1	1.96	0.27	7.19
ENST00000565382.1	2.15	0.49	4.38
ENST00000567732.1	2.54	0.94	2.69
ENST00000569087.2	4.36	1.74	2.51
ENST00000576215.1	95.15	2.23	42.61
ENST00000582866.1	3.02	1.15	2.62
ENST00000585496.1	302.20	9.97	30.31
ENST00000587088.1	269.53	12.25	22.00

4h v/s 0h

CF	FPKM at 4h	FPKM at 0h	FC
Nil			
**Non CF**	**FPKM at 4h**	**FPKM at 0h**	**FC**
ENST00000429829.5	20.70	8.39	2.47
ENST00000452120.6	12.29	4.71	2.61
ENST00000526906.1	1.13	0.51	2.19
ENST00000534918.1	15.32	7.57	2.02
ENST00000605862.4	3.45	1.59	2.17
ENST00000612517.1	2.77	1.38	2.01

6h v/s 0h

CF	FPKM at 6h	FPKM at 0h	FC
ENST00000491934.2	31.57	14.71	2.15
ENST00000517927.1	3.42	0.43	7.92
ENST00000602361.1	237.22	33.37	7.11
ENST00000623072.1	3.12	0.97	3.22
**Non CF**	**FPKM at 6h**	**FPKM at 0h**	**FC**
ENST00000434245.2	1.65	0.55	2.97
ENST00000438324.1	1.32	0.50	2.61
ENST00000491934.2	94.00	30.66	3.07
ENST00000517927.1	1.17	0.18	6.51
ENST00000595748.1	1.95	0.69	2.84

2h v/s 0h			
**Non CF**	**FPKM at 2h**	**FPKM at 0h**	**FC**
ENST00000593554.1	2.10	0.45	4.64
ENST00000595428.1	2.99	0.60	4.98
ENST00000598070.1	3.97	0.32	12.59
ENST00000599274.1	22.71	2.36	9.64
ENST00000602458.1	2.85	1.20	2.37
ENST00000602597.1	1.18	0.32	3.65
ENST00000602820.1	1.47	0.64	2.31
ENST00000602890.1	299.79	120.27	2.49
ENST00000604014.1	2.23	0.51	4.35
ENST00000606064.2	4.62	1.15	4.02
ENST00000606194.1	6.32	0.10	65.62
ENST00000607956.1	8.10	1.94	4.17
ENST00000608012.1	4.34	1.38	3.15
ENST00000609183.1	9.89	1.30	7.62
ENST00000609649.1	3.38	0.34	9.83
ENST00000610058.1	2.48	0.87	2.85
ENST00000610220.1	1.39	0.29	4.81
ENST00000612365.1	36.58	13.16	2.78
ENST00000613543.1	1.45	0.33	4.41
ENST00000614061.1	2.03	0.86	2.35
ENST00000614912.1	2.91	0.51	5.69
ENST00000616815.1	43.27	10.02	4.32
ENST00000617652.1	2.61	0.41	6.43
ENST00000618070.1	1.95	0.81	2.42
ENST00000619432.1	5.39	1.22	4.42
ENST00000624421.1	3231.21	155.92	20.72
ENST00000624988.1	11.02	2.11	5.22
ENST00000625139.1	5.51	0.86	6.42
