# Supplementary material for: Bronchial Epithelial Cells from Cystic Fibrosis Patients Express a Specific Long Non-coding RNA Signature upon Pseudomonas aeruginosa Infection
Source: Front Cell Infect Microbiol. 2017 May 29;7:218. doi: 10.3389/fcimb.2017.00218 (PMC5447040; doi:10.3389/fcimb.2017.00218)
Supplement: Supplementary file 5 [file Table5.PDF]

Supplementary Table 5. LncRNA transcripts Downregulated at 2h,4h,6h in comparison with 0h timepoints in CF and non CF groups

**2h v/s 0h**			
**CF**	**FPKM at 2h**	**FPKM at 0h**	**FC**
ENST00000429368.1	0.47	1.07	0.44
**Non CF**	**FPKM at 2h**	**FPKM at 0h**	**FC**
Nil			

**4h v/s 0h**			
**CF**	**FPKM at 4h**	**FPKM at 0h**	**FC**
ENST00000445184.1	0.75	1.73	0.43
**Non CF**	**FPKM at 4h**	**FPKM at 0h**	**FC**
ENST00000569087.2	0.96	1.92	0.50

**6h v/s 0h**			
**CF**	**FPKM at 6h**	**FPKM at 0h**	**FC**
ENST00000331856.6	0.34	1.01	0.34
ENST00000420195.1	0.75	1.78	0.42
ENST00000440570.5	4.05	9.31	0.44
ENST00000449500.1	1.24	2.55	0.49
ENST00000451937.5	39.05	93.19	0.42
ENST00000499842.1	0.53	1.16	0.46
ENST00000561486.1	1.32	3.54	0.37
ENST00000565162.2	0.57	1.17	0.49
ENST00000606034.1	0.89	2.33	0.38
ENST00000609755.1	1.01	2.27	0.45
ENST00000623593.1	1.73	4.26	0.41
ENST00000625139.1	0.37	1.17	0.31
ENST00000626538.1	0.56	1.25	0.45
**Non CF**	**FPKM at 6h**	**FPKM at 0h**	**FC**
Nil			
