# Supplementary material for: Bronchial Epithelial Cells from Cystic Fibrosis Patients Express a Specific Long Non-coding RNA Signature upon Pseudomonas aeruginosa Infection
Source: Front Cell Infect Microbiol. 2017 May 29;7:218. doi: 10.3389/fcimb.2017.00218 (PMC5447040; doi:10.3389/fcimb.2017.00218)
Supplement: Supplementary file 6 [file Table6.PDF]

Supplementary Table 6: Fold changes of lncRNA transcripts (illustrated in Figure 3B) which are differentially up-regulated (FC>2) at 2, 4 and 6 h vs 0 h, exclusively in non-CF cells with their values from CF/non CF analysis.

Transcript ID	Gene ID	FC in CF/non-CF			FC at different time points *vs* 0h		
		2h	4h	6h	2h *vs* 0h	4h *vs* 0h	6h *vs* 0h
ENST00000360656.2	CH17-340M24.3	0.21	1.13	1.31	7.13	0.91	0.84
ENST00000411579.1	CTD-3184A7.4	0.31	0.73	0.66	3.07	1.13	0.79
ENST00000416221.5	RP11-295G20.2	0.30	1.08	1.01	2.64	0.80	0.81
ENST00000422971.1	LINC01589	0.18	0.98	1.34	3.80	0.66	0.61
ENST00000424948.1	RP1-92O14.3	0.26	0.80	1.11	4.52	1.37	1.21
ENST00000429630.1	AC093673.5	0.53	1.18	1.27	2.29	0.88	0.78
ENST00000430034.1	AC132217.4	0.12	1.26	2.17	18.50	0.69	0.90
ENST00000444464.1	AC084809.3	0.34	1.12	1.41	3.76	0.90	0.99
ENST00000446211.1	PARD3-AS1	0.17	1.01	1.03	5.50	1.48	1.18
ENST00000469903.5	RP11-706O15.1	0.29	1.05	1.40	3.59	0.96	0.82
ENST00000473970.2	RPARP-AS1	0.23	0.91	0.99	4.19	0.67	0.69
ENST00000504573.1	CTB-129O4.1	0.25	0.50	0.50	5.37	1.81	1.47
ENST00000528887.1	AP003068.9	0.04	1.27	1.77	22.42	0.68	0.59
ENST00000531523.1	SNHG9	0.16	0.82	1.14	6.43	1.24	0.63
ENST00000537269.1	U47924.27	0.33	1.45	1.22	4.14	0.78	0.70
ENST00000563192.1	SNHG19	0.30	0.75	0.78	3.70	1.23	0.79
ENST00000564248.1	RP3-406A7.7	0.21	0.91	0.94	4.01	0.85	0.86
ENST00000565152.1	RP11-388M20.1	0.18	1.12	0.61	7.19	0.84	0.84
ENST00000565382.1	RP11-505K9.1	0.26	0.79	1.08	4.38	1.15	0.69
ENST00000567732.1	CTA-14H9.5	0.55	1.13	1.32	2.69	1.15	1.26
ENST00000569087.2	RP5-1085F17.3	0.35	1.03	1.36	2.51	0.50	0.76
ENST00000576215.1	CTD-2561B21.7	0.02	0.43	0.19	42.61	0.79	0.77
ENST00000582866.1	RP11-498C9.15	0.37	0.89	1.27	2.62	0.69	0.88
ENST00000585496.1	CTD-2659N19.10	0.05	1.53	1.88	30.31	0.61	0.82
ENST00000587088.1	CTD-2369P2.4	0.03	0.89	0.91	22.00	0.67	0.52
ENST00000593554.1	CTD-2192J16.26	0.20	0.79	0.59	4.64	1.30	0.69
ENST00000595428.1	LA16c-OS12.2	0.18	0.77	0.83	4.98	1.02	0.56
ENST00000598070.1	AC005339.2	0.13	0.61	1.41	12.59	1.44	3.07
ENST00000599274.1	CTC-246B18.10	0.04	1.23	0.61	9.64	0.62	0.64
ENST00000602458.1	RP11-95D17.1	0.42	0.92	0.91	2.37	0.87	0.97

Transcript ID	Gene ID	FC in CF/non CF			FC at different time points *vs* 0h		
		2h	4h	6h	2h *vs* 0h	4h *vs* 0h	6h *vs* 0h
ENST00000602597.1	RP11-849F2.9	0.28	0.54	1.01	3.65	1.20	0.74
ENST00000602820.1	RP11-738E22.3	0.29	0.82	1.41	2.31	0.83	0.76
ENST00000602890.1	RP11-3P17.5	0.36	1.09	1.18	2.49	0.80	0.71
ENST00000604014.1	RP11-385F7.1	0.33	0.95	1.82	4.35	0.89	0.52
ENST00000606064.2	RP11-722E23.2	0.20	0.82	1.43	4.02	0.64	0.92
ENST00000606194.1	CTD-2256P15.4	0.02	1.06	1.98	65.62	0.61	0.60
ENST00000607956.1	RP11-33O4.1	0.24	1.00	1.11	4.17	0.93	1.13
ENST00000608012.1	RP11-1275H24.3	0.33	0.72	0.81	3.15	1.06	1.13
ENST00000609183.1	RP11-434H6.7	0.10	1.02	1.11	7.62	0.63	0.68
ENST00000609649.1	RP4-597N16.4	0.07	1.33	1.54	9.83	0.55	0.47
ENST00000610058.1	RP11-127B20.2	0.19	0.72	1.09	2.85	0.44	0.71
ENST00000610220.1	RP11-73K9.3	0.23	1.87	2.14	4.81	0.65	0.80
ENST00000612365.1	RP5-875H18.9	0.35	1.06	1.10	2.78	0.81	0.85
ENST00000613543.1	RP11-324E6.10	0.24	0.59	1.23	4.41	1.36	0.89
ENST00000614061.1	RP5-890E16.5	0.45	0.95	0.94	2.35	0.89	1.08
ENST00000614912.1	RP11-321A17.6	0.19	1.09	0.44	5.69	0.70	0.87
ENST00000616815.1	AP001505.10	0.25	1.02	1.19	4.32	1.00	0.77
ENST00000617652.1	RP11-1055B8.9	0.15	0.74	0.43	6.43	1.59	1.52
ENST00000618070.1	RP11-248M19.1	0.45	0.73	1.01	2.42	1.21	0.78
ENST00000619432.1	RP11-670E13.6	0.34	1.17	1.21	4.42	1.32	1.35
ENST00000624421.1	AC005943.6	0.04	0.94	0.87	20.72	0.70	0.71
ENST00000624988.1	RP11-2K6.1	0.14	1.35	1.29	5.22	0.49	0.89
ENST00000625139.1	RP11-477I4.4	0.14	0.63	0.76	6.42	1.01	0.51
ENST00000429829.5	XIST	4.84	1.16	1.06	0.57	2.47	2.06
ENST00000452120.6	MEG3	4.48	1.95	3.11	0.65	2.61	1.34
ENST00000526906.1	RP11-351I24.1	0.70	0.63	0.70	1.52	2.19	1.60
ENST00000534918.1	CTD-2228K2.7	1.41	0.91	1.23	0.95	2.02	1.25
ENST00000605862.4	RP11-274B21.14	1.05	0.69	0.87	1.03	2.17	1.66
ENST00000612517.1	RP11-676J12.9	0.72	0.67	0.59	1.31	2.01	1.46
ENST00000434245.2	RP11-495P10.8	0.21	0.07	0.05	0.76	2.00	2.97
ENST00000438324.1	RP5-1185I7.1	0.63	0.61	0.22	0.62	1.36	2.61
ENST00000595748.1	CTC-490G23.2	0.35	0.36	0.21	1.18	1.31	2.84

Please Note: The highlighted values are upregulated with calculated Fold change (FC) after the filter FPKM>1. The values not highlighted, but FC more than 2 should not have satisfied the FPKM filter.
