# Supplementary material for: Bronchial Epithelial Cells from Cystic Fibrosis Patients Express a Specific Long Non-coding RNA Signature upon Pseudomonas aeruginosa Infection
Source: Front Cell Infect Microbiol. 2017 May 29;7:218. doi: 10.3389/fcimb.2017.00218 (PMC5447040; doi:10.3389/fcimb.2017.00218)
Supplement: Supplementary file 7 [file Table7.PDF]

Supplementary Table 7: Fold changes of lncRNA transcripts (illustrated in Figure 3A) which are differentially up-regulated (FC>2) at 2, 4 and 6 h vs 0 h, exclusively in CF cells with their values from CF/non CF analysis.

ENS IDs	FC in CF/non-CF			FC at different time points *vs* 0h		
	2h	4h	6h	2h *vs* 0h	4h *vs* 0h	6h *vs* 0h
ENST00000602361.1 (Syn: RMRPR, NME1, CHH, RRP2)	1.60	1.13	3.11	6.32	0.32	7.13
ENST00000623072.1 (CTC-444N24.7)	1.36	1.24	1.83	1.69	1.26	3.22

Please Note: The highlighted values are upregulated with calculated Fold change (FC) after the filter FPKM>1. The values not highlighted, but FC more than 2 should not have satisfied the FPKM filter.
