# Supplementary material for: Bronchial Epithelial Cells from Cystic Fibrosis Patients Express a Specific Long Non-coding RNA Signature upon Pseudomonas aeruginosa Infection
Source: Front Cell Infect Microbiol. 2017 May 29;7:218. doi: 10.3389/fcimb.2017.00218 (PMC5447040; doi:10.3389/fcimb.2017.00218)
Supplement: Supplementary file 8 [file Table8.PDF]

Supplementary Table 8: Fold changes of lncRNA transcripts (illustrated in Figure 3A) which are differentially down-regulated (FC<0.5) at 2, 4 and 6 h vs 0 h, exclusively in CF cells with their values from CF/non CF analysis.

Transcript ID	Gene ID	FC in CF/non CF			FC in CF at different time points *vs* 0h		
		2h	4h	6h	2h *vs* 0h	4h *vs* 0h	6h *vs* 0h
ENST00000429368.1	MEG9	2.66	2.89	3.24	0.44	1.01	0.76
ENST00000445184.1	LINC01004	1.60	0.69	0.81	1.03	0.43	0.61
ENST00000331856.6	RP11-69E11.4	1.18	0.69	0.76	0.57	0.57	0.34
ENST00000609755.1	RP11-1246C19.1	1.04	0.82	0.72	0.71	0.71	0.45
ENST00000606034.1	RP11-54O7.17	1.12	1.11	0.71	0.81	0.80	0.38
ENST00000449500.1	CTD-3184A7.4	0.66	0.61	0.67	0.70	0.51	0.49
ENST00000440570.5	MIR503HG	0.43	1.10	0.90	0.72	0.54	0.44
ENST00000565162.2	LINC01311	0.63	0.73	0.77	0.83	0.68	0.49
ENST00000451937.5	MIR205HG	1.01	0.76	0.62	0.84	0.69	0.42
ENST00000626538.1	BLACAT1	0.93	0.58	0.48	0.89	0.53	0.45
ENST00000625139.1	RP11-477I4.4	0.14	0.63	0.76	0.75	0.51	0.31
ENST00000623593.1	RP11-1334A24.5	2.80	1.36	1.28	0.87	1.08	0.41
ENST00000420195.1	SH3BP5-AS1	0.92	0.70	0.68	0.73	0.65	0.42
ENST00000499842.1	RP11-1094H24.4	1.25	0.83	0.94	0.78	0.58	0.46
ENST00000561486.1	RP11-448G15.3	1.25	0.76	0.63	0.79	0.70	0.37

Please Note: The highlighted values are downregulated with calculated Fold change (FC) after the filter FPKM>1. The values not highlighted, but FC more than 2 should not have satisfied the FPKM filter.
